# Characterization of four rice *UEV1* genes required for Lys63-linked polyubiquitination and distinct functions

**DOI:** 10.1186/s12870-017-1073-8

**Published:** 2017-07-17

**Authors:** Qian Wang, Yuepeng Zang, Xuan Zhou, Wei Xiao

**Affiliations:** 1 College of Life Sciences, Capital Normal University, Beijing, 100048 China; 2 Department of Microbiology and Immunology, University of Saskatchewan, Saskatoon, SK S7N 5E5 Canada

**Keywords:** Rice, Uev1, Ubc13, Lys63-linked polyubiquitination, DNA-damage response, Prenylation

## Abstract

**Background:**

The error-free branch of the DNA-damage tolerance (DDT) pathway is orchestrated by Lys63-linked polyubiquitination of proliferating cell nuclear antigen (PCNA), and this polyubiquitination is mediated by a Ubc13-Uev complex in yeast. We have previously cloned *OsUBC13* from rice, whose product functions as an E2 to promote Lys63-linked ubiquitin chain assembly in the presence of yeast or human Uev.

**Results:**

Here we identify four highly conserved *UEV1* genes in rice whose products are able to form stable heterodimers with OsUbc13 and mediate Lys63-linked ubiquitin chain assembly. Expression of *OsUEV1*s is able to rescue the yeast *mms2* mutant from death caused by DNA-damaging agents. Interestingly, OsUev1A contains a unique C-terminal tail with a conserved prenylation site not found in the other three OsUev1s, and this post-translational modification appears to be required for its unique subcellular distribution and association with the membrane. The analysis of *OsUEV1* expression profiles obtained from the Genevestigator database indicates that these genes are differentially regulated.

**Conclusions:**

We speculate that different OsUev1s play distinct roles by serving as a regulatory subunit of the Ubc13-Uev1 complex to respond to diverse cellular, developmental and environmental signals.

## Background

Ubiquitination is a critical post-translational protein modification process in eukaryotic cells, which involves a small protein modifier named ubiquitin (Ub). Although ubiquitination is well known to target proteins for degradation [1, 2], several non-proteolytic roles have also been found including manipulating protein interaction, activities and localization [3–5]. Different fates of the target protein after ubiquitination are often dictated by whether it is monoubiquitinated, or additional ubiquitins are attached to form a poly-Ub chain. In the latter case, the C-terminus of an incoming Ub can be linked to one of seven surface lysine residues (Lys6, Lys11, Lys27, Lys29, Lys33, Lys48 and Lys63) on the previous Ub [3, 6]. It was found that different poly-Ub chains have different topological and chemical properties; for example, while Lys11, Lys29 and Lys48 linked chains lead to protein degradation [2, 3, 7], the Lys63-linked chain is generally involved in signal transduction [5].

Ubiquitination was initially implicated in DNA-damage response when Rad6, an E2 enzyme, was found to be required for post-replication repair (PRR) in budding yeast [8]. Rad6, along with its cognate E3 Rad18, monoubiquitinates proliferating cell nuclear antigen (PCNA) at the Lys164 residue in response to replication-blocking DNA damage; this monoubiquitination leads to translesion DNA synthesis (TLS). The monoubiquitinated PCNA can be further polyubiquitinated at the same residue by the E2-E3 complex Mms2-Ubc13-Rad5 [9, 10], which is required for error-free lesion bypass [11–14] via template switch [15, 16]. This process appears to be conserved in eukaryotic organisms from yeast to human, and is named DNA-damage tolerance (DDT) [17, 18].

Owing to their sessile nature, plants are continuously under different types of stresses, such as DNA damage by UV exposure. These stresses severely compromise plant survival, reduce crop yield and threaten food security. Plants have established several strategies to cope with DNA-damage stresses, including various DNA repair pathways and tolerance of replication blocks by efficient TLS polymerases [19–24]. Meanwhile a few reports also indicate the conservation of error-free DDT in *Arabidopsis* [25–27]; however, little is known about the underlying mechanisms. We previously reported the cloning and characterization of rice *UBC13*, a putative error-free DDT gene, and showed that it is able to functionally complement the corresponding yeast *ubc13* mutant’s defect in PRR, and its product mediates Lys63-linked polyubiquitination in vitro [28]. In both cases, rice Ubc13 has to rely on a heterologous Ubc-E2 variant (Uev). Indeed, Ubc13 and Uev proteins from yeast or mammalian cells form a stable heterodimer, which is absolutely required for Lys63-linked poly-Ub chain assembly [29–31], and this process appears to be highly conserved in eukaryotes [18]. In this study, four rice *UEV1* genes are identified and functionally characterized. Interestingly, one of the four rice *UEV1* products, Uev1A, is deemed to be post-translationally modified in its C-terminus, which makes it functionally different from other three Uev1s, suggesting that they are involved in multiple cellular processes, that they have distinct functions and that rice Uevs may serve as a regulatory subunit to modulate Ubc13 activities.

## Results

### The rice genome encodes four highly conserved *UEV1* genes

Our previous work has identified the *UBC13* gene in rice, which is predicted to produce a protein which is highly conserved with Ubc13s from some other species [28]. In general, Ubc13 works with Uev as a heterodimer to catalyze the assembling of Lys63-linked Ub chains, and OsUbc13 was proved to be able to interact with Uevs from yeast and human to achieve this goal. Therefore, it is reasonable to predict that the rice genome contains its own conserved *UEV* gene(s). In this study, the *Arabidopsis UEV1A* gene was used to BLAST the rice genome in the Rice Annotation Project Database (RAP-DB, http://rapdb.dna.affrc.go.jp/index.html). Four genes were retrieved and named *OsUEV1A* (Os03g0712300, GenBank accession number XM_015777395.1), *OsUEV1B* (Os12g0605400, XM_015764791.1), *OsUEV1C* (Os09g0297100, XM_015756494.1) and *OsUEV1D* (Os04g0684800, XM_015780422.1). The exon-intron organization and coding sequences of these rice loci were determined through sequence comparison with the PCR-amplified corresponding full-length cDNAs and available sequences from RAP-DB. Based on the cDNA PCR products detected, all the *OsUEV1* cDNA products were identical to corresponding annotations on RAP-DB.

**Fig. 1:**
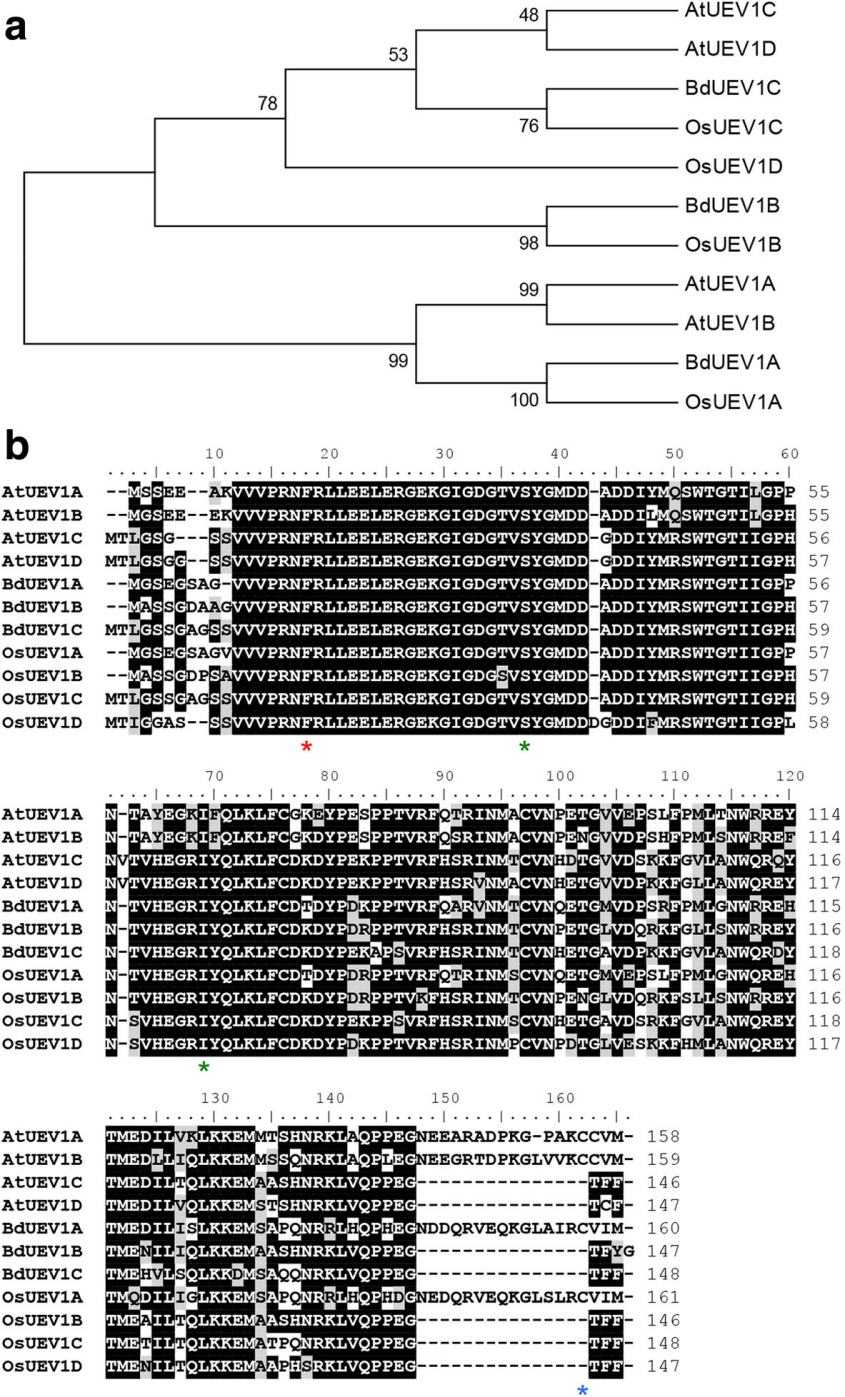
*OsUEV1* phylogenetic and sequence analyses. **a** Phylogenetic analysis among OsUev1s, AtUev1s and BdUev1s. The tree is built by MEGA6 software. **b** Protein sequence alignment of OsUev1s, AtUev1s and BdUev1s. The sequences were processed using the BioEdit program v7.0.9. Identical residues are highlighted in black while conserved residues are in grey. Asterisks indicate known functional residues defined in yeast and human Uevs

Phylogenetic analysis was performed on the ORF sequences of *OsUEV1*s, *Arabidopsis thaliana* (*At*) *UEV1*s [26] and *Brachypodium distachyon* (*Bd*) *UEV1*s [32] (Fig. 1a), which reveals that *OsUEV1A* is evolved from the same *UEV* ancestor as *AtUEV1A, AtUEV1B* and *BdUEV1A*, while the other three *OsUEV1*s are closely related to *AtUEV1C, AtUEV1D, BdUEV1B* and *BdUEV1C*, suggesting that they were duplicated and further evolved within each species. Of particular interest is that *OsUEV1*s are more closely related to their respective *BdUEV1* partners than *AtUEV1*s, consistent with a notion of parallel evolution within monocotyledon and dicotyledon plants.

Based on the ORF sequences of all four *UEV1* genes from rice, *OsUEV1B*, *OsUEV1C* and *OsUEV1D* are predicted to encode proteins with 146, 148 and 147 amino acids, respectively, whereas the predicted OsUev1A protein contains 161 amino acids with a C-terminal extension, which was also found in AtUev1A, AtUev1B and BdUev1A (Fig. 1b). It was noted that all plant Uev1s with the C-terminal extension contain a conserved CaaX motif predicted to be a target of prenylation, a protein lipid modification that facilitates the protein-protein or protein-membrane interaction by attaching the isoprenoid groups (a 15-carbon farnesyl or 20-carbon geranlygeranyl) to the Cys residue (blue asterisk) [33, 34]. In addition, several critical residues implicated in Uev activity are also conserved among all Uev1s, including hMms2-F13 (red asterisk) known to be required for the physical interaction with Ubc13 [29], and hMms2-S32 and I62 (green asterisks) required for non-covalent interaction with Ub and poly-Ub chain assembling [35, 36] (Fig. 1b).

### OsUev1s physically interact with OsUbc13 to form a stable heterodimer

**Fig. 2:**
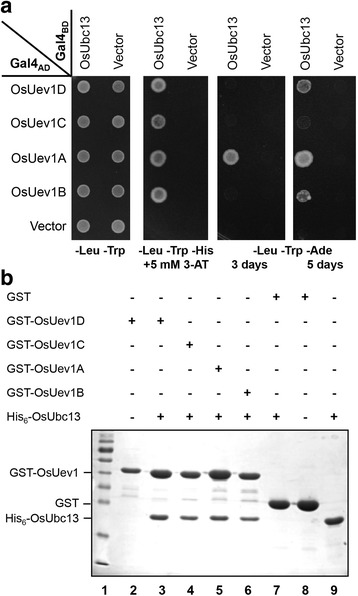
Physical interaction between OsUev1s and OsUbc13. **a** Physical interaction between OsUev1s and OsUbc13 by a yeast two-hybrid assay. PJ69-4A transformants carrying one Gal4_AD_ (from pGAD424 derivative) and one Gal4_BD_ (from pGBT9 derivative) were replicated onto various plates as indicated and incubated at 30 °C for 3 (or 5, as indicated) days before being photographed. **b** Protein interactions between OsUev1s and OsUbc13 by an affinity pull-down assay. *E. coli* BL21 cells transformed with GST-OsUev1D (Lane 2), GST (Lane 8) and His_6_-OsUbc13 (Lane 9) alone served as controls. pGEX6 (GST) co-transformed with His_6_-OsUbc13 (Lane 7) also served as a negative control. Lane 1 contains prestained molecular weight markers (SM0661, Fermentas). Lanes 2–8 contain samples from the GST affinity pull-down products while Lane 9 contains purified His_6_-OsUbc13. The SDS-PAGE gel was stained with Coomassie blue

Lys63-linked polyubiquitination is thought to regulate target proteins in a non-proteolytic manner, and Ubc13 is the only known E2 dedicated to mediating Lys63-linked polyubiquitin chain assembly. However, the prerequisite of this activity is that Ubc13 must be associated with a Uev to form a stable heterodimer [30, 31]. To test whether the four predicted rice Uev1s function in a similar manner, we first assessed their ability to interact with OsUbc13 by a yeast two-hybrid assay. Indeed all four OsUev1s are able to interact with OsUbc13, as none of the negative controls are able to grow under same experimental conditions (Fig. 2a). However, the strength of association appears to be different among OsUev1s; in the high-stringent -Ade medium, OsUev1A grows better than other three OsUev1s (Fig. 2a).

To further confirm the direct interaction between OsUbc13 and OsUev1s, we performed an in vitro glutathione *S*-transferase (GST) pull-down assay with recombinant proteins purified from *Escherichia coli.* Indeed, all GST-tagged recombinant OsUev1s are able to pull-down His_6_-tagged OsUbc13, while as a control, GST protein alone fails to do so (Fig. 2b). From the above observations, we conclude that all four OsUev1s are able to interact with OsUbc13 directly and form stable heterodimers.

### OsUev1s are required for Lys63-linked polyubiquitin chain assembly in vitro by OsUbc13

**Fig. 3:**
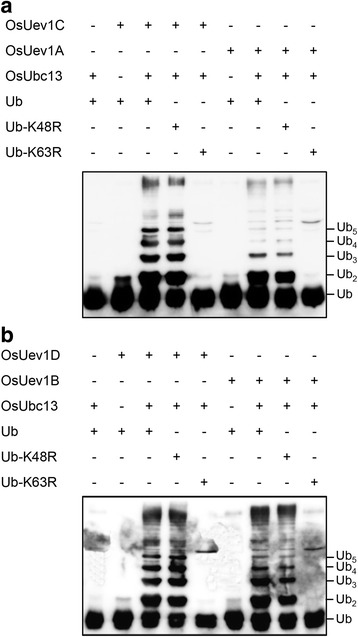
OsUev1s and OsUbc13 promote K63-linked poly-Ub chain assembly. After in vitro ubiquitin conjugation reactions as described in Methods, samples were subjected to Western blotting analysis using an anti-Ub antibody to monitor free poly-Ub chain formation. All reactions contain E1 and Mg-ATP, while OsUbc13, OsUev1 and Ub in each reaction are as indicated in the upper panel. Ub and different lengths of Ub chains are marked. **a** OsUev1A and OsUev1C with OsUbc13. **b** OsUev1B and OsUev1D with OsUbc13

We previously reported that OsUbc13 is a functional E2 capable of assembling Lys63-linked Ub chains along with yeast Mms2 or human Uev1A in vitro [28]. In *Arabidopsis*, both Ubc13A and Ubc13B are also able to promote Lys63-linked poly-Ub chain formation in the presence of Uev1s [26]. To test whether these four OsUev1s are biochemically active, we performed an in vitro ubiquitination assay with recombinant OsUbc13 and OsUev1s. As shown in Fig. 3, OsUbc13 or any one of the OsUev1s alone cannot trigger the Ub chain formation in the presence of E1 and ATP in the reaction buffer, but OsUbc13 with any one of the OsUev1s can generate free poly-Ub chains. Furthermore, to determine which kind of Ub chain linkage the E2 complexes assemble, we utilized site-specific Ub-Lys mutations. As shown in Fig. 3, each OsUbc13-OsUev1 complex is still able to mediate poly-Ub chain assembly with Ub-K48R but not with Ub-K63R, confirming that the Ub chains are linked through Lys63. It is noted that among the four OsUev1s, OsUev1A primarily promotes the di-Ub chain formation and its ability to promote poly-Ub chains is relatively weaker than other three OsUev1s (Fig. 3a, b).

### Functional complementation of yeast *mms2* by *OsUEV1* genes

**Fig. 4:**
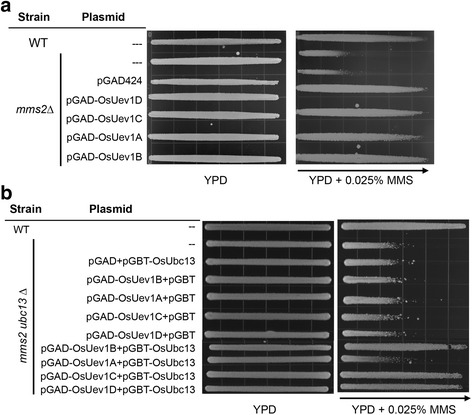
Functional complementation of yeast single and double mutants. **a** Functional complementation of the *mms2* single mutant by *OsUEV1* genes. WXY902 (*mms2∆*) transformants were incubated overnight and printed onto YPD and YPD + MMS gradient plates. The plates were incubated at 30 °C for 2 days before being photographed. Only one selected MMS-containing plate is shown. The arrow indicates increased MMS concentration. **b** Functional complementation of the *mms2∆ ubc13∆* double mutant (WXY955) by *OsUBC13* along with *OsUEV1s*. Experimental conditions were as described in **a**

To test whether *OsUEV1*s are functionally conserved between different species, we performed a DNA-damage sensitivity assay to determine whether *OsUEV1*s could functionally complement the error-free DDT defect in a yeast *mms2* null mutant. As shown in Fig. 4a, expression of any one of the *OsUEV1* genes is capable of rescuing the *mms2* null mutant from death caused by treatment with methyl methanesulfonate (MMS) to a level comparable with wild-type cells, whereas *mms2* null mutant cells carrying the empty vector are not rescued, indicating that OsUev1s are functionally conserved with yeast Mms2 and likely able to form a heterodimer with yeast Ubc13.

Since *OsUBC13* is also able to complement the yeast *ubc13* null mutant [28], we next asked whether the OsUbc13-OsUev1 complexes are able to functionally complement the yeast *mms2 ubc13* double mutant. The yeast *mms2 ubc13* double mutant cells were co-transformed with two yeast plasmids expressing *OsUBC13* and *OsUEV1*, or a corresponding empty vector. As expected, neither *OsUBC13* nor *OsUEV1*s with corresponding empty vectors is able to rescue the yeast *mms2 ubc13* double mutant. Surprisingly, while the combination of *OsUBC13* with *OsUEV1B*, *OsUEV1C* or *OsUEV1D* restored the yeast *mms2 ubc13* double mutant sensitivity to MMS to the wild-type level, the combination of *OsUBC13* and *OsUEV1A* did not provide *mms2 ubc13* mutant cells with MMS resistance (Fig. 4b).

### Roles of the OsUev1A C-terminal domain and its putative prenylation site

**Fig. 5:**
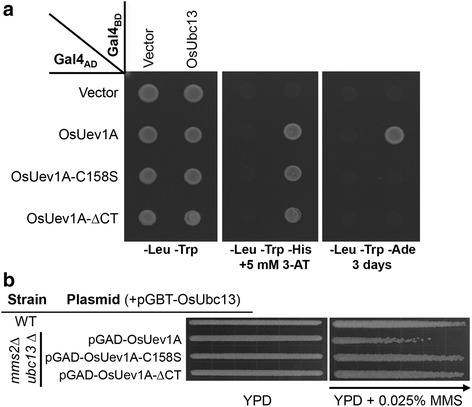
Effects of OsUev1A C-terminal domain and the putative prenylation site on the Ubc13-Uev1 complex formation and functional complementation in yeast. **a** Yeast two-hybrid analysis of physical interaction between OsUbc13 and OsUev1A or its derivatives. Experimental conditions were as described in Fig. 2a. **b** Functional complementation of the yeast *mms2∆ ubc13∆* mutant by OsUev1A and its derivatives in the presence of pGBT-OsUbc13. Experimental conditions were as described in Fig. 4

To ask whether the C-terminal extension in general or the prenylation motif in particular is responsible for the above observed distinct phenotypes of OsUev1A over other OsUev1s, we made two OsUev1A-derived constructs, namely OsUev1A-∆CT that removes the C-terminal 18 amino-acid tail and OsUev1A-C158S in which the conserved C158S amino acid substitution prevents potential prenylation [33, 34]. In a yeast two-hybrid assay (Fig. 5a), both OsUev1A mutant derivatives reduced the interaction capacity with OsUbc13 in comparison to OsUev1A, since they were unable to grow in the high-stringent -Ade plate [37]. Meanwhile, together with *OsUBC13*, the two *OsUEV1A* mutant derivatives restored the MMS resistance in the yeast *mms2 ubc13* mutant strain (Fig. 5b), reminiscent of *OsUEV1B*, *OSUEV1C* and *OsUEV1D*. From the above observations, we conclude that the C-terminal tail of OsUev1A and its putative prenylation is responsible for its unique phenotypes in yeast cells.

### The C-terminal tail and putative prenylation of OsUev1A determines its subcellular localization and membrane association

**Fig. 6:**
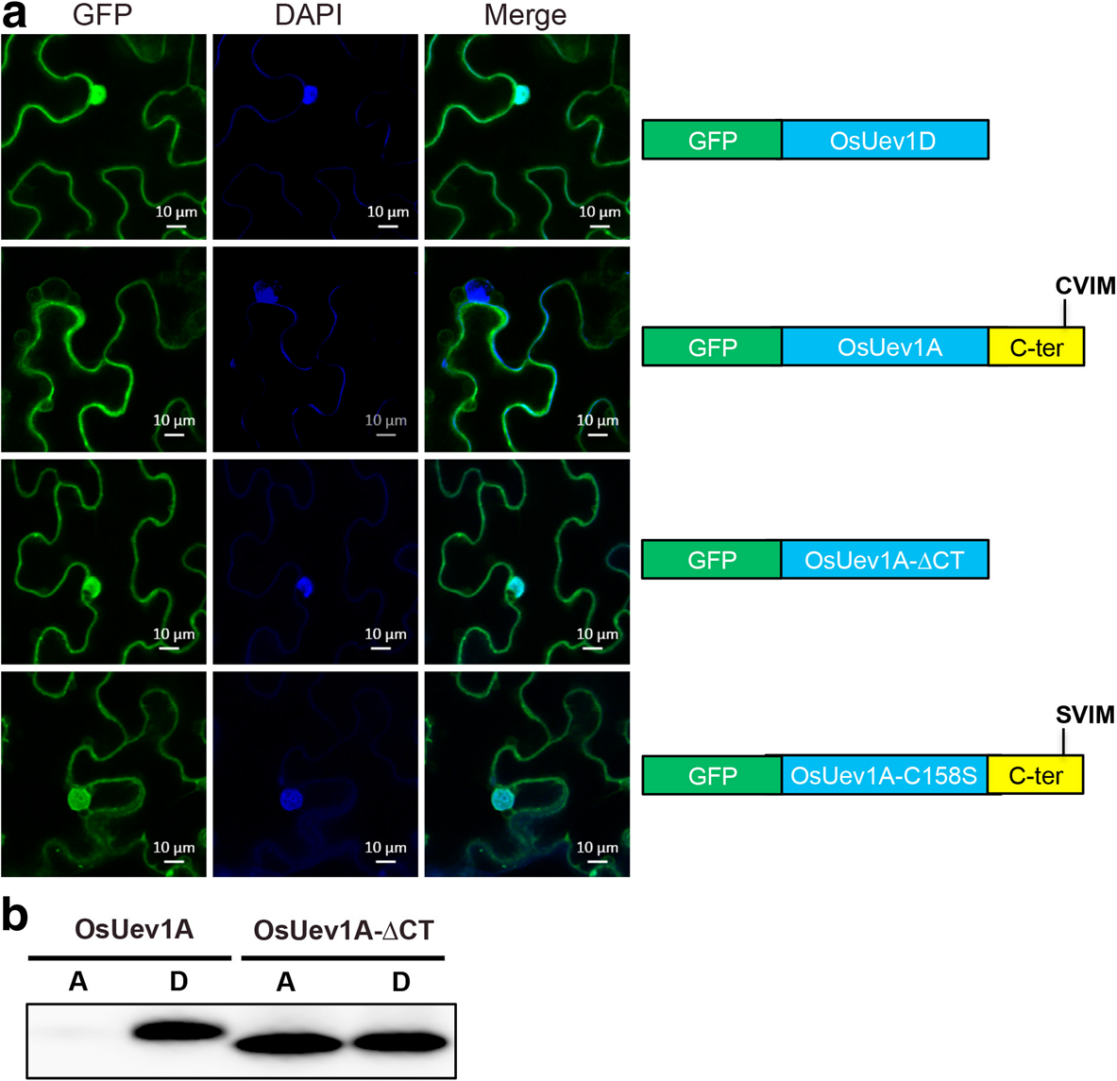
Subcellular distribution of selected OsUev1s. **a** Subcellular localization of GFP-OsUev1s and their derivatives as illustrated in the right panel. The GFP-tagged OsUev1s were expressed in *N. benthamiana* leaves by an *Agrobacterium*-mediated infiltration method. Photos were taken after 2–3 days of infiltration. A representative image is shown for each transformant. **b** The above transformed *N. benthamiana* leaves were analyzed by a protein-partitioning assay as described in Methods. GFP-OsUev1A and GFP-OsUev1A-∆CT were detected by an anti-GFP antibody (B-2, sc-9996, Santa Cruz). A: aqueous phase; D: detergent phase

To further understand cellular functions of OsUev1s, we monitored the subcellular localization of selected OsUev1s in *Nicotiana benthamiana* leaves. In this experiment, GFP-fused OsUev1D is found in both cytoplasm and nucleus (Fig. 6a, top row). In contrast, GFP-OsUev1A is clearly excluded from the nucleus (Fig. 6a, 2nd row). As all four OsUev1s are highly conserved in their core region except that OsUev1A contains an additional C-terminal tail, we asked whether the unique localization pattern of OsUev1A is caused by its C-terminus. As shown in the third row of Fig. 6a, after removal of its C-terminal tail sequence, the subcellular localization pattern of GFP-OsUev1A-∆CT appears to be different from that of OsUev1A and comparable to that of OsUev1D, particularly for the nuclear localization. Furthermore, GFP-OsUev1A-C158S behaves like GFP-OsUev1A-∆CT and differs from OsUev1A (Fig. 6a, 4th row). These observations indicate that the C-terminal tail and most likely the prenylation of OsUev1A is responsible for its subcellular distribution.

Since protein prenylation has been reported to facilitate protein-protein interaction and/or protein-membrane interaction [33, 34], we asked whether GFP-tagged OsUev1 variants are indeed associated with the membrane. Tobacco leaves transformed with GFP-tagged OsUev1 variants were subject to a Triton X-114 based protein-partitioning assay. As shown in Fig. 6b, GFP-tagged OsUev1A is almost exclusively found in the detergent (D) phase, whereas C-terminally truncated GFP-OsUev1A is partially diffused to the aqueous (A) phase. The partial dissociation of Os-Uev1A-∆CT from membrane fraction is because either it still contains another membrane association motif, or the ectopically expressed GFP-tagged OsUev1 level is higher than the native Uev1A. Nevertheless, these results collectively indicate that OsUev1A preferentially associates with membrane and that this association is dependent on its C-terminal sequence and probably on its prenylation, whereas other three OsUev1s are soluble proteins spread in both cytoplasm and the nucleus.

### Expression of *OsUEV1*s in different tissues and during different developmental stages

**Fig. 7:**
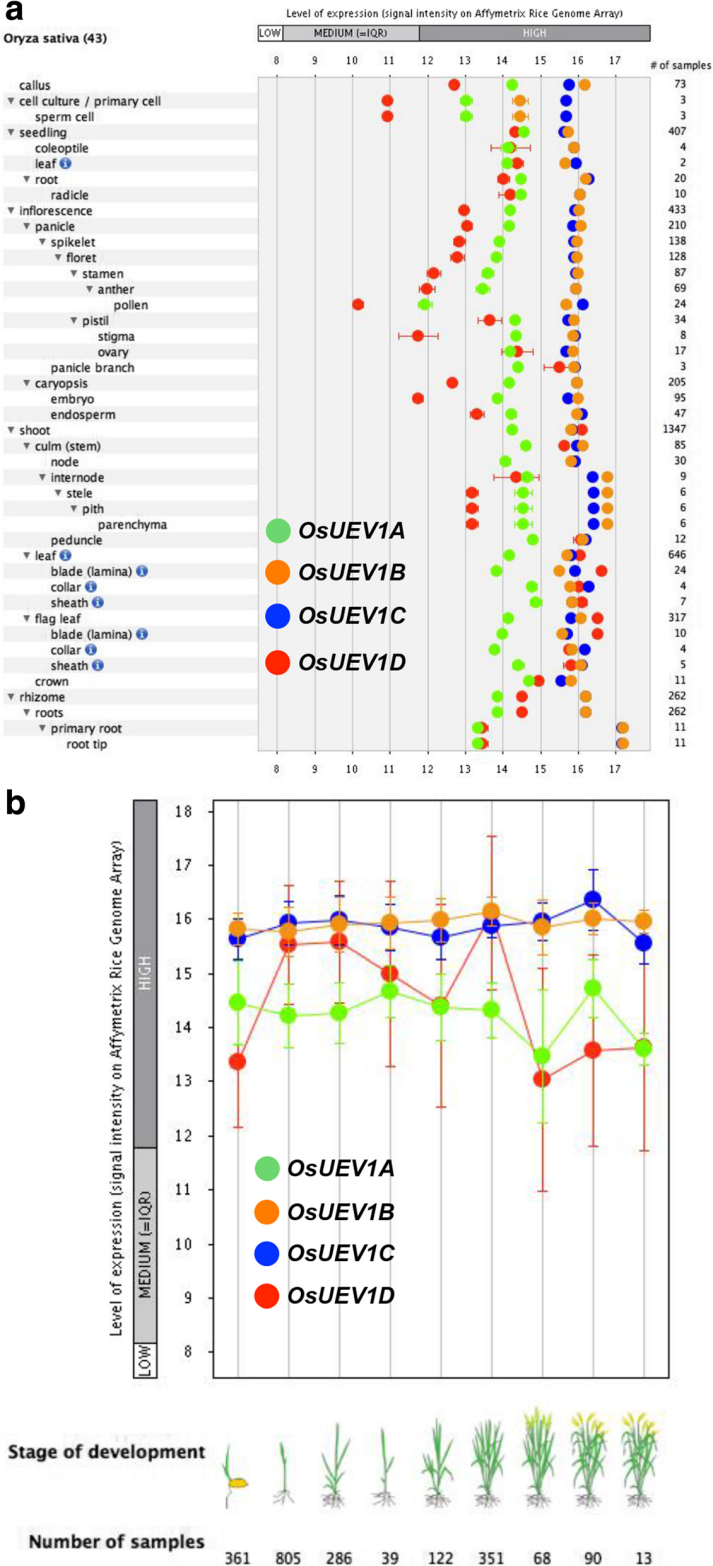
Quantitative analysis of *OsUEV1* expression. **a** Expression of *OsUEV1A*, *OsUEV1B*, *OsUEV1C* and *OsUEV1D* in different tissues. Samples were taken from different tissues as indicated and relative transcript levels of the entire transcriptome were determined by microarray analysis. **b** Expression of the four *OsUEV1* genes during different life stages. The above data were retrieved from Genevestigator (www.genevestigator.com)

Since we have previously shown that the expression of *UBC13* both in *Arabidopsis* [25] and rice [28] remains constitutive in different tissues and even under stresses, we speculated that *OsUEV1*s, like their *Arabidopsis* counterparts [26], may be regulated at the transcriptional level to modulate the Ubc13-Uev complex activity. We searched microarray databases online by utilizing Genevestigator. The retrieved data as shown in Fig. 7a indicate that the four *OsUEV1*s display various expression patterns in different tissues. Both *OsUEV1B* and *OsUev1C* maintain a constant and relatively high-level expression in different tissues, whereas the expression of *OsUEV1A* and *OsUEV1D* fluctuates rather dramatically*.* For example, OsUEV1D is expressed at very high level in various parts of the leaf, but its expression is extremely low in pollen and sperm cells. During rice development, *OsUEV1B* and *OsUev1C* still maintain a stable and high-level expression, while the expression of *OsUEV1A* is also stable but the transcript level is relatively low. In contrast, the expression of *OsUEV1D* fluctuates dramatically during development (Fig. 7b).

**Fig. 8:**
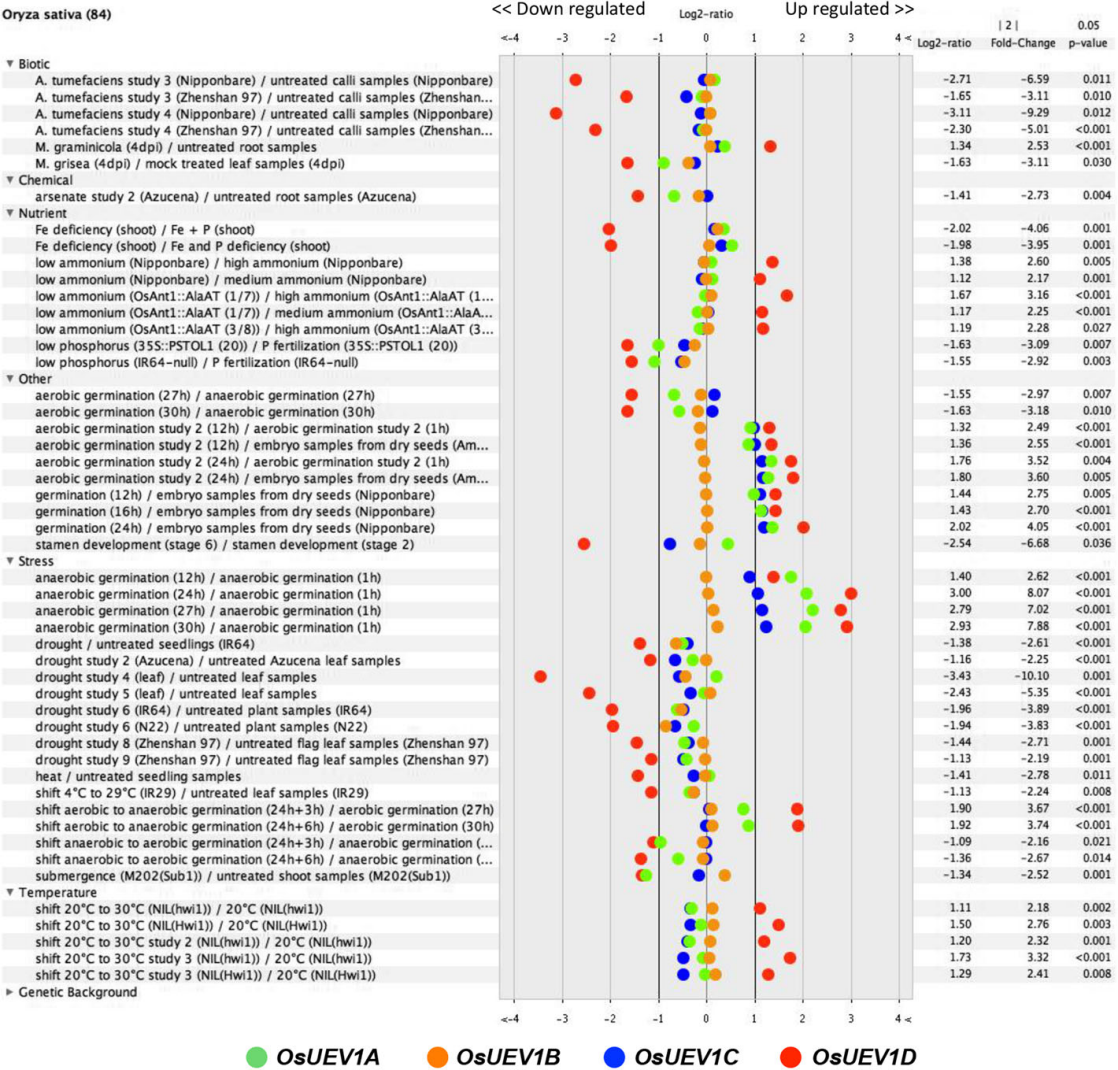
Expression of *OsUEV1*s in response to various perturbations. Samples were taken from different treatments as indicated and relative transcript levels of the entire transcriptome were determined by microarray analysis. The data were retrieved from Genevestigator (www.genevestigator.com) and only significant results (change ≥ twofold) are shown

We also analyzed *OsUEV1* expression patterns in response to various environmental stresses as reported from the database (Fig. 8), in which the expression of *OsUEV1B* and *OsUEV1C* is remarkably constant. In contrast, *OsUEV1D* is highly sensitive to essentially all perturbations examined. For example, its expression appears to be repressed under drought conditions and highly induced during anaerobic seed germination. Interestingly, *OsUEV1D* is induced when seeds are shifted from aerobic to anaerobic conditions for germination, while its expression is repressed when seeds are shifted from anaerobic to aerobic germination conditions. The expression of *OsUEV1A* is also perturbed in response to various biotic and abiotic stresses to moderate extents, most notably during anaerobic seed germination.

## Discussion

In this study, we identified and cloned four highly conserved *UEV* genes from the rice genome and our in vitro studies confirm that these Uevs are able to interact with OsUbc13 to form a stable heterodimer and mediate Lys63-linked polyubiquitination. Functional studies indicate that these rice *UEV* genes can restore cellular activity of the yeast *mms2* null mutant for resistance to a DNA-damaging agent, reminiscent of the ability of *OsUBC13* to restore the corresponding yeast *ubc13* mutant [28]. Furthermore, several observations are consistent with the notion that the four *OsUEV1*s confer different functions in vivo. Firstly, when both yeast *MMS2* and *UBC13* genes are replaced by different combinations of *OsUBC13* and *OsUEV1*s, three of them can fully restore the DNA-damage tolerance activity, while *OsUBC13-OsUEV1A* cannot. Secondly, in a yeast two-hybrid assay the OsUbc13-OsUev1A interaction appears to be stronger than the other three pairs, ruling out a possibility that the lack of functional complementation by *OsUBC13-OsUEV1A* is due to reduced physical interaction. Finally, the subcellular localization of OsUev1A differs from that of OsUev1D (and presumably OsUev1B and OsUev1C) in plants. While OsUev1D behaves like a small soluble protein and appears to be enriched in the nucleus, OsUev1A is excluded from the nucleus and appears to be membrane-bound. The above observations collectively indicate that OsUev1s confer function(s) in addition to DDT and that different OsUev1s may have distinct physiological functions. This conclusion is not unexpected as in yeast, the regulation of the DDT pathway is the only known function of Ubc13-Mms2; however, the two distinct Ubc13-Uev complexes turn out to be multi-functional in multi-cellular organisms like mammals [38]. Hence, it is reasonable to speculate that Uevs are also multi-functional in plants. Indeed, plant Ubc13 has been implicated to function in apical dominance [39], iron metabolism [40], innate immunity [41] and auxin signaling [42], and at least some of the above functions may require the Ubc13-Uev E2 complex and Lys63-linked ubiquitination.

Mammalian genomes contain one *UBC13* gene and at least two *UEV* genes, and the *UEV* genes often confer distinct functions. For example, mammalian Ubc13 regulates the DDT pathway by interacting with Mms2 and mediates NF-κB signaling by associating with Uev1A [38]. In this study, we identified four *OsUEV* genes in rice and at least one of them, *OsUEV1A*, functions differently from other *OsUEV* genes. Similar results are also observed in *Arabidopsis*, in which AtUev1A and AtUev1B contain additional C-terminal sequences [26]. A novel finding in this study is that OsUev1A distributes differently in the cellular compartment than other OsUev1s, and that its unique localization and membrane-bound property can be abolished by removal of the C-terminus or simply mutating the predicted prenylation site. Although exactly which cellular role(s) it plays remains unknown, it can be cautiously predicted based on this study that it is membrane-related and non-nuclear. This function must be critical for the plant development and/or environmental response, as essentially all known plant genomes contain at least one OsUev1A ortholog with predicted CaaX motif at their C-terminus (data not shown). On the other hand, the remaining three OsUev1s may function in DNA-damage response like their *Arabidopsis* Uev1D counterparts [26], consistent with the observed OsUev1D nuclear localization. Given that AtUbc13 has been implicated in several cellular processes and these functions are likely conserved with OsUbc13, it is of great interest to investigate which Ubc13-mediated cellular process involves which OsUev1 and to discover additional cellular processes in which Ubc13-Uev participates.

As a non-canonical ubiquitination, Lys63-linked ubiquitination is most likely involved in stress response signaling, where Ubc13-Uev plays a critical role in assembling Lys63-linked poly-Ub chains on the target protein. Therefore, it is conceivable that its activity is tightly regulated in response to different environmental signals. To date, no report has found altered activity of Ubc13 in plant species examined [25, 28, 43]. Instead, its activity and specificity are largely determined by the cognate Uev, and the cellular levels of Uev appear to fluctuate in different tissues and in response to various environmental stresses [44–46]. Furthermore, the pathway involvement of Lys63-linked Ub chain is mainly determined by the Uev that interacts with Ubc13 [38]. In this study, four distinct *UEV* genes in rice also display different expression patterns among different tissues, life stages and environmental stresses. In addition to the constitutively expressed *OsUEV1B* and *OsUEV1C* genes, the *OsUEV1A* and *OsUEV1D* expression fluctuates under all the above conditions, suggesting that these two gene products play regulatory roles under different biological processes. Hence, the regulation of Uev activity appears to be evolutionarily preferred and Uevs serve as regulatory subunits of the Ubc13-Uev E2 complex in response to distinct cellular and environmental signals.

## Conclusions

In this article, we report the molecular cloning and functional characterization of four rice *UEV1* genes. Like other plant species, rice also contains two classes of *UEV1* genes with their encoded proteins differ in the C-terminal extension. This study reveals that OsUev1A contains a C-terminal tail not found in other three OsUev1s, that the tail sequences are highly conserved within higher plants, from both monocotyledon and dicotyledon, and that a putative posttranslational modification site is also conserved. Our limited experimental results showed that the two classes of *OsUEV1*s genes function differently in a heterologous yeast host and that their protein subcellular distribution patterns are also different in plants. Furthermore, the above differences are attributed to the OsUev1A C-terminal tail and most likely to its putative prenylation. Unlike the *OsUBC13* gene that is constitutively expressed, database analyses reveal that the expression of four *OsUEV1* genes fluctuates dramatically in different tissues, during different developmental stages as well as in response to various biotic and abiotic stresses, suggesting that these *OsUEV1* gene products regulate the Ubc13-Uev1 activity.

## Methods

### Plant materials and yeast cell culture

Rice (*Oryza sativa* L. cv. Japonica) seeds were surface sterilized with 2% NaClO for 30 min after a pre-wash by sterile distilled water, followed by washing seven times in sterile water. The sterilized rice seeds were plated in Murashige and Skoog (MS) plates containing 2.2 g/l minimal organics, 10 g/l sucrose and 1% agar. They were cultured in a growth chamber (16 h light/8 h dark and 30 °C).

Yeast strains used in this study include PJ69-4A [37] for the yeast two-hybrid assay, HK578-10D (*MATa ade2–1 can1–100 his3–11,15 leu2–3, 112 trp1–1 ura3–1*) and its *mms2Δ::HIS3* derivative WXY902 and *mms2Δ::HIS3 ubc13Δ*::*hisG-URA3-hisG* derivative WXY955 for the functional analysis. Yeast cells were grown at 30 °C in either rich YPD or a synthetic dextrose (SD) medium supplemented with nutrients as instructed [47]. To make plates, 2% agar was added to YPD or SD medium prior to autoclaving. Yeast cells were transformed by a LiAc method [48].

### Cloning rice *UEV1* cDNAs and plasmid construction

To clone the full-length *OsUEV1* open reading frames (ORFs), total RNA was extracted from rice seedlings with TRIzol reagents (Invitrogen, Carlsbad), which was used as a template for RT-PCR using the RevertAid First Strand cDNA Synthesis Kit (Fermentas). Gene-specific primers are as follows: *OsUEV1A*: 5′-taaccg*gaattc*ATGGGGTCCGAGGGATC-3′ and 5′-ggcacgc*gtcgac*TTACATGATGACACACCTA-3′; *OsUEV1A-C158S*: 5′-ggcacgc*gtcgac*TTACATGATGACAC**T**CCTA-3′; *OsUEV1A-∆CT*: 5′-ggcacgc*gtcgac*TTAGCCATCATGGGGTTGATG-3′; *OsUEV1B*: 5′-gaaccg*gaattc*ATGGCGTCGAGTGGAGAT-3′ and 5′-gcacgc*gtcgac*CTAGAAGAATGTCCCCTC-3′; *OsUEV1C*: 5′-tgaccg*gaattc*ATGACGCTGGGGAGCTC-3′ and 5′-gcacgc*gtcgac*CTAGAAGAACGTCCCTTC-3′; *OsUEV1D*: 5′-taactg*gaattc*ATGACGATCGGCGGCG-3′ and 5′-tcccgc*gtcgac*CTAGAAGAAGGTCCCTTC-3′. The forward primers contain the *Eco*RI restriction site and the reverse primers contain the *Sal*I site, as italicized. The PCR product of *OsUEV1A-1D* ORFs were cloned into a yeast two-hybrid vector pGAD424Bg, which was derived from pGAD424 [49].

### Yeast two-hybrid analysis

The yeast two-hybrid strain PJ69-4A [37] was used for this assay. The co-transformation, selection and two-hybrid detection steps were as previously described [28].

### Recombinant protein purification and ubiquitination assay

*OsUEV1* ORFs were isolated from pGAD-OsUev1s and cloned into pGEX6p-1. The resulting pGEX-OsUev1s were transformed into *E. coli* BL21 CodonPlus (DE3)-RIL cells. The pGEX-OsUev1 fusion proteins were purified following a previously published protocol [38]. Meanwhile, GST and His_6_-OsUbc13 were produced and purified as previously described [28]. For an in vitro ubiquitination assay, a previously described protocol [28] was followed.

### GST pull-down assay

The *E. coli* BL21 competent cells were transformed with either pGEX6p-1, pGEX-OsUev1s alone, or co-transformed with pET-OsUbc13. The whole-cell extracts were incubated with Glutathione Sepharose 4B Microspin™ beads (17–0756-01, GE Healthcare) at 4 °C for 2 h, which were then harvested by centrifugation, washed 5 times with a lysis buffer and boiled with 2 × loading buffer. The products were analyzed on a 12% SDS-PAGE gel.

### Yeast gradient plate assay

Yeast strain HK578-10D and its isogenic *mms2∆* single or *ubc13∆ mms2∆* double mutants were either singly transformed with pGAD-OsUev1A-1D or co-transformed with pGAD-OsUev1s and pGBT-OsUbc13. The transformants were selected on SD-Leu (for *mms2∆*) or SD-Leu-Trp (for *ubc13∆ mms2∆*) plates. The gradient plate assay was conducted as described [50].

### Subcellular localization

The ORFs of *OsUEV1*s and derivatives were amplified and cloned into the pCAMBIA1302 vector containing an N-terminal GFP tag. These *GFP-OsUev1*s constructs were transformed into the *Agrobacterium tumefaciens* (GV3101/pMP90), and positive colonies were cultured overnight and infiltrated into *Nicotiana benthamiana* leaves as described [51]. After 2–3 day incubation, epidermal cells of the transformed tobacco leaf were viewed by confocal microscopy (Zeiss LSM 780, Germany). Excitation parameters are 488 nm and 405 nm for GFP and DAPI, respectively.

### Protein partitioning assay

For protein partitioning assay, total protein was extracted from transformed *N. benthamiana* leaves using a buffer containing 50 mM Tris-HCl pH 8.0, 0.3 M NaCl, 1% TritonX-114, 10 mM PMSF, 3 mM DDT and 1 tablet (for 50 ml buffer) protease inhibitors (Roche). The extract was incubated in Triton X-114 containing buffer for 1 h at 4 °C before centrifugation at 12,000 *g* for 10 min at 4 °C. Samples were then incubated at 37 °C for 5 min and centrifuged at 12,000 *g*. The aqueous upper phase and detergent-enriched lower phase were separated and extracted once again with detergent and aqueous solutions, respectively. The resulting four samples were adjusted to equal volume and proteins were precipitated with chloroform/methanol prior to Western blot analysis.
